# “If you weren't connected to the Internet, you were not alive”: experience of using social technology during COVID-19 in adults 50+

**DOI:** 10.3389/fpubh.2023.1177683

**Published:** 2023-10-09

**Authors:** Katrina Ling, Danielle Langlois, Harrison Preusse, Jennifer M. Rheman, Danya Parson, Sarah Kuballa, Martin Simecek, Katherine M. Tsui, Marlena R. Fraune

**Affiliations:** ^1^Intergroup Human-Robot Interaction Lab, Department of Psychology, New Mexico State University, Las Cruces, NM, United States; ^2^Faculty of Informatics, Masaryk University, Brno, Czechia; ^3^Toyota Research Institute, Cambridge, MA, United States

**Keywords:** older adults, social technology, loneliness, COVID-19, mental health

## Abstract

**Introduction:**

Loneliness and social isolation reduce physical and mental wellbeing. Older adults are particularly prone to social isolation due to decreased connection with previous social networks such as at workplaces. Social technology can decrease loneliness and improve wellbeing. The COVID-19 pandemic prompted quarantine and social distancing for many people, creating a context of widespread social isolation.

**Method:**

In the current study, we interviewed middle-aged and older adults' (n = 20) about their use of social technology when social isolation was common: during the early part of the pandemic while social isolation and masking were still required in the United States, between August 2020 and June 2021.We analyzed the data using three-phase coding. We compare our results against the model of the bidirectional and dynamic relationship between social internet use and loneliness.

**Results:**

We found that during the COVID-19 pandemic, our participants experienced decreased social interaction and moved toward online interaction. Participant use of social technology supported the stimulation hypothesis - that is, they used it to maintain existing relationships and social connection. The findings also add novel evidence that the stimulation hypothesis endures for older adults during enforced isolation (in this case due to the COVID- 19 pandemic).

**Discussion:**

Based on our data, we also propose adding the presence or realism of connection via social technology as a main factor to the model and engaging with construal level theory of social presence to fill in critical variables of this relationship. We further find that digital exclusion acts as a barrier to obtaining benefits from stimulation via social technology and recommend that further research examined digital exclusion in relation to the bidirectional and dynamic model. Finally, we discuss recommendations for improving social technology to benefit middle-aged and older adults.

## 1. Introduction

As adults age, they experience significant changes in family structure and companionship as children leave the home, they leave the workplace, or as older friends and family members pass away (2, 3). This often leads to loneliness, depression, health concerns, and an overall decrease in quality of life (4–7).

Many people across ages are lonely and have limited access to social connection for various reasons. In addition to old age, this can include distance from family members, disability, and socioeconomic status (for example, not having access to a car). One potential solution is social technology: devices or applications that facilitate humans to socially interact with each other (8). When persons are isolated, social technology can increase their access to community, decrease feelings of loneliness, and improve their overall wellbeing (9).

However, many older adults lack access to these social technologies or have difficulty using them effectively (8, 10). Qualitative research examining what older adults need from social technology in recent years is scant, but necessary, because social technologies have changed dramatically, and it is important to understanding how older adults are using or not using social technology (10).

In the current study, we explored older adults' subjective experiences with current social technology to better understand their challenges with it, with the goal of improving older adults' ability to use social technology to connect with others and decrease loneliness. We use these data to explore when social technology use stimulates vs. displaces social interaction connection (the stimulation and displacement hypotheses of social technology use). We assessed how the three levels of digital exclusion (access to technology, skills to use technology, and tangible benefits from technology) affected older adults' experience with technology.

### 1.1. Loneliness

#### 1.1.1. Social isolation and loneliness

Social isolation is a lack of social connections or lack of emotional closeness with one's social connections, and it leads to increased loneliness as well as various negative physical and mental health outcomes (11, 12). Loneliness is the negative affective state people experience when they perceive themselves as having insufficient social connection (13). Health consequences correlated with loneliness include increased morbidity and mortality, decreased mental health and cognitive function, increased smoking and drinking, and increased risk of depression (7, 14, 15).

#### 1.1.2. Loneliness in older adults

Loneliness is prevalent in older adults, as children leave the home, and as older friends and family members pass away (2, 3). Further, they may have decreased mobility, lack of socioeconomic resources, and may not have access to previous social avenues such as workplaces (4, 5). Often, their children have left home, and associated social connections, such as with their child's school and extracurricular groups may also decrease, which often results in loneliness and decreased satisfaction (16) Social isolation among older adults is especially a wellbeing concern because they are at higher risk than younger populations for autoimmune, cardiovascular, neurocognitive, and emotional wellness issues. In older adults, social isolation and chronic loneliness relate to increased doctor visits due to poor health, high blood pressure, increased rates of mental illness, and increased mortality (4–7, 15, 17, 18).

#### 1.1.3. Loneliness and the COVID-19 pandemic

While social connection and isolation are important issues in general, they became particularly pertinent during the COVID-19 pandemic. A virus formally known as SARS-Cov-2 was first detected in December of 2019. By March of 2020, many countries began to take public health measures to contain the spread of the virus. At the beginning of data collection for the study in August 2020, more than 10 million people in the world had been infected by the virus (19), a year later, in September 2021, there were over 218 million confirmed cases of COVID-19 and over 4.5 million deaths due to COVID-19 (19). To reduce the spread of this virus, national and international institutions ordered quarantine—that is, physical distance and isolation—in most countries around the world (20).

During this time, many individuals led to people avoiding in-person socialization, as schools, restaurants, pubs, offices, and shops were mandated to close (21, 22). Public health organizations advocated for “social distancing,” which refers to a variety of behaviors intended to stop the spread of the disease, including maintaining a 6 ft (2 m) distance between individuals, and limiting in-person contact with persons not in one's household (23). Because self-isolation and social distancing became necessary due to COVID-19, it is also important to consider the negative impacts it can have on people's health (17). Due to COVID-19 restrictions and social distancing guidelines, older adults were at a higher risk of feeling lonely and isolated as compared to before the COVID-19 pandemic, due to precautions implemented to stop the spread of the virus (5).

The COVID-19 pandemic has increased avoidance of older adults (5); this is in part due to evidence that older adults are more likely to have severe complications and die from the illness (24, 25). Additionally, people often stereotype older adults as being a burden, dependent, and sickly, which elicits avoidance toward and neglect of the older adults (4). Individuals who follow public health guidelines and maintain distance from older adults to “keep them safe” may cause increased social isolation for older adults (5). Because previous research has found correlations between loneliness and various health repercussions (22, 26) it is critical to find ways to improve social connection for older adults. This is important both during and after the pandemic because of the prevalence of loneliness in older adults (4, 5) and because increasing social connection can improve health. Further, this pandemic gave the opportunity to study what happens when older adults experience sudden new reasons for this isolation, and these findings extend beyond new isolation due to a pandemic (e.g., due to a new health problem).

### 1.2. Social technology

As individuals strive to maintain vital social connections during physical isolation, social technology has become more prevalent and crucial than before. Social technology is any technology that facilitates social interaction between humans, including but not limited to telephone, email, text, social media, and video call (8). According to Nowland's bidirectional and dynamic model of the relationship between social Internet use and loneliness (1), social technology can reduce or increase loneliness, depending on if their use is to stimulate social connection (stimulation hypothesis) or to disengage from the real world (disengagement hypothesis).

#### 1.2.1. Social technology as a potential solution to loneliness

In the stimulation hypothesis, people can use technology to support existing relationships and create new ones (27, 28). Use of social technology in these ways to communicate with others is correlated with decreased loneliness and increased wellbeing (29–31). Social technology has played an important role in facilitating social interaction, specifically in older adults who want to maintain contact with their loved ones. Use of social technology between friends, family, and companions correlates with an increased feeling of social support in older adults (9, 32, 33). Older adults' comfort with technology under everyday circumstances increased the likelihood of using those technologies under stressful situations, such as global pandemics (2).

#### 1.2.2. Possible negative effects of social technology

In the disengagement hypothesis, technology can displace real-world relationships, leading to loneliness. Passive social media use, consumption of social media without active social engagement (34) or using the Internet to disengage from the real world shows no benefits to health (35–37). Overstimulation from technology during stressful times can lead to feeling stressed and overwhelmed. Research has linked negative mental health with exposure to media on pandemics, which suggests that avoiding the behavior of seeking out stressful media content can protect our psychological wellbeing (38). Additionally, if persons have access to a technology, but are not able to use it well, they cannot benefit from its use (8, 39).

#### 1.2.3. Older adults and social technology

Older adults make up one population that is often considered to be “digitally excluded” (40). Digital exclusion is not as straightforward as whether one can or cannot use technology (40, 41), but occurs at three levels: access to technology, skills to use technology, and tangible benefits from outcomes of the Internet, including economic, social, political, and educational (42–45).

While older adults are sometimes stereotyped as unwilling to use technology, many older adults hold largely positive attitudes toward social technology (8). One reason for negative attitudes toward certain technology is that some researchers and practitioners have developed technology “for older adults” based on stereotypes of older adults as frail or housebound, which limits older adults' abilities. Conversely, whereas many older adults prefer to maintain their autonomy and are more interested in technology that helps them accomplish their goals (46). Instead of developing technology for older adults based on assumptions, it is critical to work with older adults to learn more about their technology needs and desires (47, 48), which we do in this paper.

Older adults may lean more toward technology that they are more accustomed to, rather than newer forms of technology popular with younger populations (49). During the pandemic, they often chose to use such technology, preferring methods of communications such as writing letters or phone calls to methods novel to them, such as video calls (50). Besides lack of access, some barriers to using social technology that older adults experience are lack of perceived self-efficacy and fears regarding privacy and security (10).

Research on older adults (aged 55+) tends to support the stimulation hypothesis (1). Due to various life changes such as relocation, health limitations, and distance from loved ones, older adults may experience a decrease in social connection, but social technologies can assist them in maintaining contact with existing social networks (3, 10). Social technology can also reduce social isolation in older adults (32, 33). More frequent social technology use related to fewer mental and physical health problems and higher self-perceptions of wellbeing in older adults (8), and older adults with higher affinity for technology had better group connection and mental health during the beginning of the pandemic (51).

However, as of yet there is little research whether the stimulation or disengagement hypothesis of social technology use will be more strongly supported during different types of loneliness or causes, such as transitory vs. chronic loneliness (1) and during times of forced social isolation, such as due to a pandemic, health issues, retirement, or difficulty traveling.

### 1.3. Current study

Social technology can help improve social connection and overall wellbeing for socially isolated individuals – but only if practitioners implement it based on the actual needs and wants of the population geared toward digital inclusion and to stimulate social connection. To this end, we conducted a qualitative interview study exploring middle-aged and older adults' subjective experiences of and challenges with using social technology. Qualitative studies of this kind are critical to understanding middle-aged and older adults' relationship with social technology because they provide rich data about user experience and suggest insights not accessible in quantitative studies (10, 52, 53).

We expanded on previous research findings that social technology correlates with a sense of social connection under the stimulation hypothesis (9, 27, 28, 32, 33). We gathered and analyzed rich information about how older adults used technology during the beginning of the COVID-19 pandemic, and their subjective feelings on how they used social technology to keep connected with their family, friends, and companions. We explored older adults' social lives before and during the pandemic, the degree to which the change affected them, and how technology could help them decrease loneliness during social isolation. To investigate this, we conducted semi-structured one-on-one interviews about how social technology can support wellbeing in middle-aged to older age adults.

Three main research questions guided this study:

- What are middle-aged and older adults' subjective experiences of using technology to socialize?
- What do older adults see as the benefits and challenges of social technology?
- How has the COVID-19 pandemic impacted middle-aged and older adults' experiences with social technology?

## 2. Materials and methods

This study was approved by the New Mexico State University Institutional Review Board.

### 2.1. Participants

Participants were 20 adults residing in the United States during the COVID-19 pandemic. We used a sample size of 20 because in qualitative samples saturation—that is, when new information tends to significantly diminish (54, 55) —typically occurs after 9 to 17 interviews (56).

Participants were required to be age 50 or older, a U.S. resident, and be able to speak English. Ages ranged from 50 to 70 (*M* = 57.6; *SD* = 6.15). Participants were recruited through a prior online mTurk questionnaire [about the Internet and social connection during the pandemic (57)], a survey recruitment agency Usertesting.com, online forums (Facebook), and snowball sampling using text describing the study. We compensated participants with a $30 USD Amazon e-gift card.

We collected data between August 2020 and June 2021; during this period, social distancing guidelines prevented many forms of in-person gatherings and traditional avenues for social connection. We use this pandemic as a time of widespread social isolation so we could interview many people who recently became more socially isolated.

Participant demographics are in Table 1.

**Table 1:** Participant demographics.

**Participant pseudonym**	**Age**	**Gender**	**Ethnicity**
Lehan	50	M	Indian
Owen	50	M	Caucasian
Caroline	51	F	Caucasian
Ian	51	M	Caucasian
Qadira	52	F	Arab-American
Grace	53	F	Caucasian
Hannah	54	F	Caucasian
Theresa	54	F	Caucasian
Patricia	55	F	Caucasian
Rebecca	55	F	Caucasian
Mark	57	M	Caucasian
Adam	58	M	Caucasian
Beverly	58	F	Caucasian
Katherine	60	F	Caucasian
David	62	M	Caucasian
Fred	64	M	Caucasian
Nate	64	M	Caucasian
Sam	65	M	Caucasian
Eleanor	68	F	Asian-American
James	70	M	Caucasian

### 2.2. Procedure

The study consisted of in-depth, semi-structured, one-on-one interviews. We conducted interviews remotely over Zoom, a teleconferencing software, due to the COVID-19 restrictions on in-person interactions. Interviews lasted between 30 and 60 min. Interviews took place between October 2020 and June 2021. Interviews were conducted by two authors.

We created the interviews specifically for this study. Because we wanted this study to potentially lead toward a participatory design study, we drew on the structure from the early, interview portion of participatory design studies (58). We crafted questions based on our main research questions (see Appendix A).

During the interview, each participant answered questions about their experiences of the COVID-19 pandemic, their participation in social distancing, and how the pandemic had impacted their social life. We used an interview outline (see Appendix A) as well as maintaining reflexivity to participant contributions and concerns (59). Participants discussed which technology platforms they use to socialize, what they like and dislike about those platforms, and what difficulties or challenges they experienced when socializing via socially assistive technology. Finally, participants also answered questions regarding how quickly they would adopt new technologies and what new technological features they might want for their social lives.

### 2.3. Analysis

We transcribed the interviews, first via Zoom's transcription software, then manually corrected by research assistants.

We conducted a thematic analysis of the transcripts using a three-phase coding process (60). In three-phase coding, researchers: (1) read interview transcripts without coding, (2) re-read the transcripts to observe recurring themes and patterns, and to create a coding frame of categories (e.g., based on frequency of topics related to the research questions), and (3) examine the transcripts in detail, noting each instance of the thematic codes (60).

We generated codes using a code-defining approach (59). In a code-defining approach, multiple coders participate in the three-phase coding system. After the second step, each person shared the codes they independently generated. From discussion of these codes, we created a final coding framework. To do so, we refined the codes and developed operational definitions; we edited some that were too specific (i.e., only applied to one participant as described), removed some that were too general, and expanded on some that we felt needed subcategories. This was done via live zoom discussions. Then coders completed the third step by independently coding the remainder of the transcripts.

The two interviewers and three undergraduate research assistants completed the coding process. We used Atlas TI 9 software to complete the analysis. For the complete coding framework (see Appendix B).

We calculated inter-rater reliability using a subset of 20% of the data. Inter-rater reliability for all categories was moderate to high, above 75% (59).

Inter-rate reliability can be seen in Table 2.

**Table 2:** Inter-rater reliability.

**Coding category**	**Operational definition**	**Percent agreement**
Social life	Participants' preferences for social interactions, types of socializing, and amount of socializing	0.77
Impact on social life	The impacts of the COVID-19 pandemic on participants' social lives	0.82
Preferences for social technology	Participants' preferences for social technology	0.89
Changes in social technology use	Changes in participants' use of social technology during the COVID-19 pandemic	0.92
Benefits and challenges of social technology	Benefits and challenges that participant experienced with social technology	0.87
Total		0.85

## 3. Results

### 3.1. RQ1: what are middle-aged and older adults' subjective experiences of using technology to socialize?

We identified three themes of participants' subjective experiences of using technology to socialize: (1) Preferences regarding interacting with people via technology vs. in person, (2) Desire for a variety of social interaction types, and (3) Level of involvement participants wanted for their social technology in their everyday lives. We discuss these below. We refer to participants with pseudonyms and their ages in brackets [e.g., Sam (65)].

#### 3.1.1. Preference for interacting in person

Most participants (*n* = 14) strongly preferred interacting with their friends and family in-person, as compared to interacting via any form of social technology. Sam [65] said that the most rewarding social interactions for him were “the ones where basically I'm face-to-face with people… and we're having a conversation.” Hannah [54] expressed a strong distaste for video call socializing, saying “the Zoom (social events) really becoming a thing shut me down a little bit. Because I prefer in person, you know?” Mark [57] mentioned lack of physical affection as an issue with social technology, saying, “I'm a kind of pretty huggy person, and you just can't do that through technology.”

#### 3.1.2. Desire for variety of social interaction types

Six participants asserted that they want a variety of different types of social interaction in their lives. Lehan [50] discussed both group and one-on-one interactions, which were either in-person, via phone, or on video call, and stated that he found all of them equally rewarding: “I like all these different types of socializing, you know? Because not every day is the same.” Eleanor [68] explained, “I don't know if I can evaluate (online vs. in-person socializing) in that way, because the ways in which they are rewarding are different, are very, very different… I can't really evaluate them and compare them to each other.”

#### 3.1.3. Variety of adoption speeds

Eight participants reported that they were slower to try new technology than their friends, while six participants felt that they were early adopters. The remaining six participants felt that their adoption rate of new technology was highly context dependent. When asked if she was quicker or slower to try new technology than her friends, Grace [53] replied, “I think I'm both. It would depend on what it is. When it's for work, they make you do it, so you're quick. The social media stuff I was slow.”

#### 3.1.4. Conflicting desire for technology involvement

Three participants reported that they wanted to have low involvement of social technology in their everyday lives. Adam [58] said of phone notifications, “I usually turn them all off. I turn off every notification I can… I don't want this thing to tell me to do something. In fact, if it did tell me to do something, I would throw it.” Two participants were very attached or “plugged in.” Qadira [52] preferred to have a high level of technological involvement in her day-to-day life and expressed that she would “rather have the notification than not be notified and miss something.” However, most participants (The remaining fifteen participants) expressed both desire for low involvement and desire for high involvement, often reporting conflicted emotions or expressing wanting different levels of technology involvement in different aspects of their lives. Lehan [50] felt that high technology use was required even if it was not preferred, saying, “there is no day or no time that I am (away from it) … I have a lot of things I do with my phone you know so that's always there.” Caroline [51] said, “I curate, I will say, my notifications pretty heavily.” Hannah [54] said, laughing, “I want to be notified, but I don't want to be notified. How about that.”

### 3.2. RQ2: what do older adults see as the benefits and challenges of social technology?

Participants discussed the benefits and challenges of using social technology. Main themes of benefits participants experienced were: (1) They maintained contact with existing social networks and (2) The benefits were better during video calls than other social technology. Main themes of challenges of social technology were: (1) Aversion to or difficulty with new social technology, (2) Wanting improvements for current technology, including for (a) Internet and bandwidth issues, and (b) the drawbacks from too much screen time, and (3) Difficulty maintaining contact with individuals who have limited expertise with social technology. Participants' experiences of challenges varied, and included lack of access to high-speed internet, difficulty learning how to use new technology, and issues regarding overall screen time.

### 3.2.1. Benefits of social technology

#### 3.2.1.1. Maintain social contact

Many participants (*n* = 12) expressed some level of appreciation for social technology and reported gratitude for the technology helping them keep in touch with their existing social networks. This was the primary benefit of social technology to participants; they did not see social technology as preferable to in-person interactions. Grace [58] said, “It has a purpose… touching base with some old friends, keeping up with faraway friends, even keeping up with friends that are close.”

#### 3.2.1.2. Preference for video calls

While participants preferred in-person interaction to video calls, they also preferred video calls to other types of social technology. When discussing the benefits of video technology, Eleanor [68] said, “What I like is that, even though you are not actually here, I can see you. I can… see your face, which means that I can sort of read your expressions.”

### 3.2.2. Challenges of social technology

#### 3.2.2.1. Aversion to new social technology

Eleven participants described experiencing difficulties when trying to learn how to use new technology. Grace [53] stated that the main barrier for her in trying new technology was having “something else to learn… it's like, ‘oh, god, how much can the brain take anymore?”' Katherine [60] described challenges when having to adapt to using new technology, saying, “Well, at the beginning of the pandemic, I felt isolated because I wasn't able to use it as well as I needed to, but by forcing myself to use it, I solved that problem… I definitely felt I didn't have access. I was on the wrong side of the digital divide.” Three participants who considered themselves early tech adopters still found using new technology to be difficult. This included Caroline [51], who expressed frustration with learning how to use video call and screen share technology: “I wish I knew how to do some of these things. But I'm not going to take the time to learn.”

Hannah [54] described having an inconsistent relationship with social technology and expressed a desire to have someone else help her with it:

“…like sometimes these things will launch with no problems, and sometimes, you know, I can't make them work to save my life. I actually missed probably two (Zoom) classes in the last 2 weeks because for some reason, they just wouldn't hook up… I could use some extra help, I guess. I don't know if it's the program or me.”

#### 3.2.2.2. Want improved current platforms

When asked what new social technology they wanted in their lives, most participants *(n* = 13) reported that they did not want to acquire any new technology. Nine of these participants said that alternatively, they wanted the technology and platforms they already used to be improved. Nate [64] said, “I like to rely on like what I have as far as a phone.” Desires for improvements varied widely. David [62] expressed frustration with attempting large group calls over video conferencing software, saying, “the current technology works well with small groups; it does not work well with anything large… that's probably where the technology fails completely.” He also expressed frustration with an inability to have side conversations in group video calls, saying “There's no way to have that kind of a large group interaction (with) small group breakouts.”

##### 3.2.2.2.1. Internet and bandwidth issues

Eight participants reported that their biggest difficulty with social technology was with internet connectivity itself. Ian [51] said, “I think the biggest problems that we have with Zoom sometimes are bandwidth issues. On one side or the other, there are bandwidth problems… that will slow things down.” Beverly [58] works as a rural public defense lawyer, and she stated that it was difficult for her and her clients to access adequate internet and phone services:

“We live in a rural area, and sometimes our internet (connection) is degraded just by weather, you know, windy weather and things like that. So that's not Zoom in particular, but just internet (connection) in general… a lot of my clients do not have access to the internet… around here, phone service can be bad. The cell towers are few and far between sometimes and so it's hard to get ahold of people.”

##### 3.2.2.2.2. Drawbacks from too much screen time

Regarding overall screen time, nine participants stated that they found the amount of time looking at screens to be an issue. Lehan [50] stated that due to the COVID-19 pandemic, “I definitely say that… screen time has increased.” Rebecca [55] stated, “I did experience headaches and I think my eyesight kind of went down a little bit, from looking at the screen… but it wasn't based on COVID per se… I was looking at the screen a lot anyway.” Theresa [54] said, “Yeah, absolutely, you get fatigued by it… your brain just starts to feel foggy.” Nine other participants stated that they spent a large amount of time looking at screens, but did not view it to be an issue, as they had not experienced any negative effects from screen use. Fred [64] said, “I guess I get as much screen time as I want. So, it's not really an issue.” Eleanor [68] described having a screen-focused lifestyle, but not having problems with that:

“It's really not a problem for me, but I used to work as a programmer. All day long I stare at computer screens, then I would come home and play computer games. So, all night long and staring at computer screens it's really not a problem.”

#### 3.2.2.3. Difficulty maintaining contact with low-technology individuals

Seven participants discussed difficulty maintaining contact with others because those people lacked access to technology or lacked the ability to use that technology effectively. Beverly discussed difficulty keeping in touch with her daughter, saying “Marie doesn't like technology, and so if you don't see Marie in person, you don't see Marie.” Eleanor [68] felt that lack of access to social technology was an issue with both her mother-in-law and aunt:

“My mother-in-law has a lot of trouble just doing anything other than basic stuff on her phone. And if you're not there to show her how you did something… she doesn't retain how to use it. And … I couldn't do Zoom with (my aunt) because I don't think that she has…the pieces like the camera, the microphone. And I don't think that she's comfortable enough with technology to connect via something like Zoom.”

### 3.3. RQ3: how has the COVID-19 pandemic impacted middle-aged and older adults' experiences with social technology?

Due to the COVID-19 pandemic, many forms of in-person socializing decreased, and use of social technology increased. We asked participants about their social lives before the pandemic, how their social interactions had been impacted by the pandemic, and about changes to their use of social technology during this time. Three themes emerged: (1) Decreased social interaction during the COVID-19 pandemic, due to (a) Growing apart from family members and (b) Keeping distance from older or vulnerable relatives, whereas others felt (c) no differences due to the pandemic. They also felt a (2) Shift from in-person to online interactions, and subthemes of (a) Feeling forced to use technology, (b) Feeling the change is permanent, and (c) Using new technology for the first time.

#### 3.3.1. Decreased social interactions and relationships during COVID-19

Most participants reported that their social interaction had decreased during COVID-19 pandemic (*n* = 15). Eleanor [68] stated that her social life had changed because she “can't get together with local friends anymore.” Grace [53] said, “There's a lot of friends that I would like to see, but I don't want to endanger them. And I also have an older mom (living) with me…so (I) definitely have not been socializing.”

##### 3.3.1.1. Growing apart from family, friends, and coworkers

Participants reported growing apart from their family members (*n* = 9), friends (*n* = 11), and coworkers (*n* = 4) over the course of the pandemic. Reasons included a lack of casual interactions that usually happen in a workplace or neighborhood (*n* = 6), and that participant had difficulty making plans for social interactions (*n* = 4). Adam [58] mentioned drifting apart from people that he used to encounter casually in his workplace: “There's people that I work with, that only show up at work 2 days a week because of the pandemic. And we used to like talk almost every day, you know, we'd run into them, but… (we) just don't do that since then.” Theresa [54] discussed growing apart from the parents of her daughter's friends:

“There were a few friends that I had before, that we used to meet up for, like, park day for our kids and things like that... And that kind of all fell apart… (those were) some of the people that I connected with the closest.”

##### 3.3.1.2. Distancing from older, vulnerable adults

Keeping distant from older, more vulnerable relatives was a recurring theme. Seven participants discussed difficulties with being unable to visit their parents due to health concerns. Fred [64] said, “my mother who lives in Wisconsin. I haven't been able to see her… the pandemic has hurt that.” Beverly [58] discussed challenges communicating with her mother over video call: “I think the Zoom visits with my mom are hard because of her dementia issues... when you see her in person, you can hold her hand or pet her shoulder, the physical contact is missed.”

##### 3.3.1.3. Feeling no change

Five participants reported that the COVID-19 pandemic did not have much of an effect on their social lives. One participant's reason for this was lack of adherence to safety guidelines. Hannah [54] stated that throughout the pandemic, she was “still going to restaurants, bars, establishments, where you can still meet up and visit. It really didn't change a whole lot.” One participant, Eleanor [68], stated that her pre-COVID social life was already mostly online:

“Most of what I do for fun while socially distancing is much the same as I did before, in truth… I have friends in other parts of the country as well, so I played video games with them, and that's most of my regular activity with them. So, I was doing that actually before COVID.”

Three participants attributed this lack of negative effect to their own introversion, and to not having a very active social life before the pandemic. Beverly [58] explained that she is “kind of an introvert. I like quiet by myself activities.” Caroline [51] said, “I'm still an introvert, so (my social life) hasn't changed as much for me as probably for other people.”

#### 3.3.2. Switch to online interaction

Eleven participants reported a switch from in-person interactions to online or phone interactions, and 12 participants reported that their overall use of technology had increased. These included one-on-one interactions, small groups of friends or family members, and more formal groups, particularly churches. Theresa [54] said, “One of the things that hit hardest is that… we belong to a church membership, so all of our meetings ended up going online.” Beverly [58] talked about informal check-ins saying, “my church started doing, like, once a month Zoom meetings, just to like, say hi to everybody, and make sure people are doing okay.” Fred [64] was part of a board game meetup group, “…that met every, every Tuesday at (local university). But that got canceled because of social distancing, of course. So, then we moved to online… so we play the same the same games but online. And for me, it's not nearly as much fun doing it online, as it is in person.”

Qadira [52] felt that regular video calls with friends in other states was particularly helpful during early stages of the pandemic, when restrictions were most stringent:

“Especially in the beginning of the pandemic, … we just were not coming out at all, even taking walks and things like that. We actually set up Zoom meetings with my friends… we had maybe four or five people at a time … that would get together… That was interesting, and that was at least some way that we could see each other. And my friends were all scattered in different parts… it was nice to be able to all get together at a certain time and see each other, even though we weren't in the same towns anymore.”

David [62] felt that his social media use had increased because “everybody is trying to maintain the social contacts that used to be done in person… so there's a lot more stuff to be engaged with and respond to than there was before.”

##### 3.3.2.1. Feeling forced to use technology

One theme that emerged was that of feeling forced to use social technology. James [70] stated that due to the COVID-19 pandemic, “you can't go out, you have to use your computer.” Grace [53] discussed using video call software while working from home and said that for her social technology use, “quite a lot that changed just for us to be able to work and keep our jobs going.” Katherine [60], who works as a professor at a community college, said “Yes, I was forced to become more technologically savvy.” She also discussed lack of access to social technology and the necessity of social technology as an issue for her students:

“When the quarantine happened… all public schools willing to quarantine and lockdown. And there was a huge portion of our population on the border and on the native American reservations, who don't have broadband, they don't have internet, they don't have computers, period. They can't even get Wi-Fi for their phones, you know, suddenly there was a whole stratum of the population who were not participating, you know, who weren't living. And we went into this strange mental concept that if you weren't connected to the Internet, you are not alive… I think that's very dangerous, especially for children, to think they are not leading a valid life if they do not have access to the Internet. That's very disturbing.”

##### 3.3.2.2. Feeling the change is permanent

Of the participants whose use of technology increased, eight participants reported that they felt these changes would become permanent, especially given an increase in virtual meetings or classes, and an increase in overall remote work and telecommuting. Adam [58] taught at a university that had transitioned from in-person courses to video call lectures via Zoom. Adam said, “going to Zoom, I don't think this is going to go backwards… I think the Zoom thing for lectures is a permanent change.” Grace [53] said of video conferencing:

“For work wise, I think it's going to be around. I think that they're learning that sometimes this is a lot cheaper than flying somebody in. And I deal worldwide with people, so this is a lot easier than trying to get people together at a location, just for a couple of meetings, when you can do it this way. And I'm also seeing that sometimes when we used to pick up the phone and call that instead we're doing the video chatting at work, and so that's nice.”

##### 3.3.2.3. Using new technology for the first time

Twelve participants reported that they had begun using video call technology for the first time during COVID-19. Grace said, “I never Zoomed or did any video conferencing before COVID, never did. Never, ever, ever.” David [62] said, “prior to COVID-19, I never used Zoom, never used Discord.” Nate [64] talked about various video calling apps, saying, “there's another one on the phone… called Duo. I use that a lot for chats, visual chats. So those things I didn't really use much before COVID.”

Four participants reported that their use of social technology had not changed at all during COVID-19. Caroline [51] said of her social technology use during social distancing, that it was “almost identical. Yeah, I have not… felt or noticed that much of a difference.” Ian [51] said, “(I) used it all before… no, there was nothing new that I found myself using.”

## 4. Discussion

We conducted a qualitative interview study with middle-aged and older adults during the first 18 months of the COVID-19 pandemic, to better understand their use of social technology during this period of social isolation, the ways in which they used social technology to combat loneliness and social isolation, and to identify their challenges and frustrations with existing social technology. We found support for the stimulation over displacement hypothesis in this population and context. We also recommend including the importance of realism or presence of social technology stimulating social interaction, as well as variables related to digital exclusion. We organize our findings by theoretical finding below.

### 4.1. Social interaction decreased

Most individuals in this study had their social lives drastically altered by the COVID-19 pandemic. Most of our participants' social interactions decreased. Some participants described decreased social connection due to a decrease in casual, incidental interaction, such as passing coworkers in a hallway. As expected, many participants reported increased social isolation and loneliness during social distancing. This is very similar to what older adults experience as they age and quit work, children move out, and friends and family members pass away (2, 3). Therefore, we expect that the findings in this paper will generalize to other situations with socially isolated middle and older adults.

Along with decreased interactions, many people switched from in-person socialization to socializing via technology, and several participants transitioned from working in-person to teleworking.

### 4.2. Participants used social technology to socially connect (supporting the stimulation hypothesis)

Participants expressed gratitude for being able to keep in touch with friends and loved ones while abiding by social distancing guidelines imposed for the COVID-19 pandemic. Consistent with previous research supporting the stimulation hypothesis (27, 28, 32, 33, 61) the middle-aged and older adults in this study used technology to increase their feelings of social connection and decrease feelings of loneliness. This provides novel evidence that during sudden prolonged isolation (in this case for the pandemic), older adults use technology to stimulate and enhance their social connections, adding to Nowland et al. (1) theory of when data support the stimulation vs. displacement hypothesis.

Further, most participants felt conflicted about how involved they wanted to be with technology. This was because they wanted to be unplugged, but also did not want to miss something important. This also feeds into the stimulation hypothesis in application to this context, because participants explicitly said that they did not want to disconnect from their lives, but instead wanted to use technology to enhance connections.

Older adults primarily use technology to maintain existing relationships. Thus, the current study also strengthened previous findings that older adults primarily use social technology to maintain existing relationships, rather than to forge new ones (3, 10).

### 4.3. Older adults preferred technology that better simulated the real world

Most people strongly preferred interacting in-person. Technology that helped them feel more like they were interacting in person (e.g., video call) was better than others (e.g., text). Thus, video calls were one of the most frequently used forms of technology. Many participants began using video call technology for the first time in their lives. Participants who had used video call technology previously reported a large increase in the frequency of its use.

However, some participants strongly disliked socializing via video calls due to a preference for in-person interaction or feeling that it was not the same or as good as in-person socializing. Shortcomings of current social technology included limited physical affection and inability to socialize effectively in large groups.

These data support and extend prior findings on the importance of vision and touch and social interaction over technology. A recent study found that older adults prefer using visual communication, and one participant indicated the importance of touch (10). The present study extends these findings, confirming the importance of audiovisual communication and supports the importance of touch for improving social technology for older adults.

Although our participants did not explicitly say it, this preference for realism in online interaction shows parallels to a need for perceived presence during the interaction. Prior work shows that people feel more social connection with others through social technology when the interaction medium more closely parallels the real world – that is, social connection is stronger moving from text to audio, to audiovisual, to telepresence robot [i.e., an audiovisual robot that users can move throughout the physical space; (62–64)], to being physically present (65).

Nowland's current model of the relationship between social Internet use and loneliness accounts primarily for stimulation or displacement (with subfactors as age and loneliness) (1). We propose *presence or realism* as a new variable in the model, with increased presence or realism of the online interaction increasing the likelihood of supporting the stimulation hypothesis. Presence or realism could also relate to other psychological factors of distance identified in construal level theory (66), such as temporal distance—e.g., a quick or immediate response would show more presence and realism than minutes- or days-delayed responses, as can be seen in text communication. This also applies to the importance of immediate responses over audio or audiovisual means as opposed to lags, such as those due to poor internet connection or bandwidth. Supporting this, our participants found lags and bandwidth problems to be especially frustrating and damaging during their interaction via social technology.

Some researchers are working on technology to address some of these limitations of social technology and improve presence and realism (67), such as technology that includes physical touch (68–71). However, most participants reported that they did not want to acquire any new social technology, but instead wanted their current social technology improved so that functions more consistently and is easier to use. Researchers should consider what additions to current technology could improve social technology for older adults (see Recommendation section below).

### 4.4. Middle and older adults experience digital exclusion to connecting via social technology

In the study, the data support that many older adults are digitally excluded related to social technology on all three levels: skills, access, and tangible social benefits (42–45). Participants were evenly spread between whether they viewed themselves as slow technology adopters, early adopters, or that their adoption rate depended on context. Still, participants had varying frustrations and challenges with the social technology.

#### 4.4.1. Skills

Approximately half of participants indicated difficulty learning how to use new social technology, and others had older relatives with difficulty in learning how to use the technology. This included phones, computers, social media, video calls, and other applications. This difficulty in learning the technology supports why past research found that older adults preferred more familiar forms of social technology such as writing letters (50).

#### 4.4.2. Access

Due to unstable internet connection, insufficient bandwidth, rural place of residence, or socioeconomic status, many participants struggled to use social technology, particularly synchronous social technology like video calls or group chats. We also found that participants reported frustration with programs glitching or not working as intended. These types of glitches may be an intersection between exclusion based on skills and access – that is, some glitches or malfunctions may be things that occur because of access issues but that people could troubleshoot with the right skills. Future researchers should consider the overlap of these three levels of digital exclusion and how they may build on each other to create even greater levels of digital exclusion.

#### 4.4.3. Social benefit and long-term tangible outcomes

Of participants who stated that their technology use had changed during the COVID-19 pandemic, the majority felt that these changes would become permanent. In particular, participants expected that telecommuting would become more common, that socializing via video call with friends or loved ones who lived far away would continue, and that remote learning and education would continue to increase, particularly at the college level. Some participants were encouraged by these changes and wanted them to become permanent because of how they could connect them to people around the world. Others expressed frustration with the thought of continuing to telecommute and attend meetings remotely; given that they were frustrated with current technology, they predicted that this advance of technology would lead to further frustrations and difficulties with social interactions. Thus, the results illustrate how digital exclusion can propagate over the long-term.

This builds on the relationship between social internet use and loneliness—that is, for groups such as older adults who experience digital exclusion, they do not gain as much of the social and long-term benefit of social stimulation from using social technology. Thus, although older adults tend to follow patterns of the social stimulation hypothesis, when they do not have as much skill or access to technology, they are still not reaching their potential to benefit from social technology for social connection. Indeed, a prior study found older adults with higher affinity for technology also felt more connected during a period of social isolation (the COVID-19 pandemic) (51). This may be especially detrimental to groups that both experience digital exclusion (e.g., people with low socioeconomic status, disability, and women) and fall into age ranges (e.g., young adult) that tend to use technology for displacement. In fact, younger adults with higher affinity for technology also felt lonelier during social isolation (the COVID-19 pandemic) (51). Thus, we propose adding digital exclusion to the model of the relationship between social internet use and loneliness.

### 4.5. Recommendations for improving social technology for older adults

Many participants reported difficulty keeping in touch with people who could not learn how to use social technology, and sometimes difficulty using it themselves. User experience researchers and designers should seek to make social technologies easier to learn how to use for older adults, so they can better use them to avoid loneliness and its detrimental health effects. Decreasing user frustration with social technology has the potential to greatly improve older adults' wellbeing and overall health. Specific changes that would improve use are:

1. Make technology simple for older adults to learn, use, and recover from failures, such as following guidelines at described in Kurniawan and Zaphiris (72) and Tsui (73).
2. Improve video calls for low internet connectivity or improving internet connectivity to rural areas.
3. Create and use alternatives to sitting and looking at a screen to decrease screen fatigue.
4. Improve video calls for groups, such as allowing side conversations without the need for breakout rooms.

We also refer readers to Greenhalgh et al. (74) for a more general set of discussion topics to consider when developing new technologies.

Historically, new technology is developed for professional use and as adoption continues becomes available to consumers. Consumers can be creative with how they use technology to engage in social interactions. In the context of mobile telepresence robots (MTRs), Boudouraki et al. (75) posit that it is sufficient for the person operating the MTR to have the “ability to participate and successfully ‘gear into' everyday social interaction, vs. the academic stance of simulating high fidelity presence. In our own MTR research (76), we found that during a 7-month deployment in which Older Adults had a robot in their home, their self-reported highlights included playing hide “n” seek and entering the dining room during a dinner and surprising her family. The mobility aspect of MTRs provides additional functionality, and these examples reinforce the sentiment that MTRs can successfully be used to participate in daily social interaction. As new technologies enter the consumer market, an extension of our research could focus on how the use of technology shapes social interactions between people, not just usability.

### 4.6. Limitations and future directions

This study was conducted with 20 residents of the United States. Because government response to the COVID-19 pandemic varied, the experiences of individuals in other countries would likely vary as well. Additionally, participants in this study were residing in various US states, and local regulations varied significantly, including which businesses were allowed to operate during which time periods. This increased variability in participants' experience of COVID-19 regulations and severity of social distancing. Future studies should examine the COVID-19 pandemic experiences of older adults from other countries. They should also examine older adults in social isolation due to non-pandemic reasons.

Most participants in the study were also healthy Caucasians. Although we studied one major population that experiences digital exclusion, older adults, other groups suffering inequalities also experience further digital exclusion, including those from historically marginalized ethnic groups (77–79) lower socioeconomic status (61, 80, 81), lower educational attainment (82–84), and those with disabilities (80, 83, 85). Further, people in “double jeopardy” (86) – that is, who are part of an intersectional disadvantaged categories [e.g., low SES and a minority ethnic group (87)] – tend to have less access to technology then people with one disadvantage category (88), increased loneliness, and lower quality of life (89). People in these different categories are likely to experience different benefits from technology and different challenges (90–92). Future research should explore the intersectionality of multiple identities related to the challenges of individuals who lack access to social technology due to lower age, SES, ethnicity, and other reasons. We strongly suggest that future researchers work directly with people who are historically marginalized to determine how social technology could help reduce loneliness and improve health.

Another major limitation is that we conducted this study remotely via teleconferencing software due to COVID-19 safety regulations. All participants in this study had access to video call technology. Older adults without such access may have had very different experiences with technology in general and specifically during the COVID-19 pandemic.

## 5. Conclusion

We compared our results against Nowland et al. (1) model of the bidirectional and dynamic relationship between social internet use and loneliness. This study confirms previous findings that older adults can and do use social technology to decrease social isolation and feelings of loneliness (8, 93), supporting the stimulation hypothesis of social technology use, especially for older adults. Older adults in this study had varying experiences with social technology, including their preferences for use, degree of involvement with social technology, and experiences of difficulties learning or using social technology. Overall, they preferred using technology that increased realism and presence during online social interaction, which may be a main factor in why the social technology succeeded at helping stimulate social connection. They wanted current technology to be improved to increase that feeling, such as by reducing problems from limited bandwidth and creating more smooth opportunities for group interaction online, such as methods for allowing side conversations within a larger group.

The study also supported the stimulation hypothesis during enforced isolation during the COVID-19 pandemic and adds to Nowland et al. (1) model the connection between isolation and stimulation using social technology. Social isolation continues to be a problem for many older adults post-pandemic because of the situations and the digital exclusion they experience. To reduce loneliness and increase physical and mental wellbeing, it is important for older adults to have access to social technology which works consistently and effectively, and which is easy for them to learn how to use. We recommend future studies examine other groups that are digitally excluded in relation to age, technology use, and Nowland et al. (1) model to provide further detail on the bidirectional and dynamic relationship between social internet use and loneliness and learn how to improve the situ.

## Data availability statement

The raw data supporting the conclusions of this article will be made available by the authors, without undue reservation.

## Ethics statement

The studies involving humans were approved by the New Mexico State University Institutional Review Board. The studies were conducted in accordance with the local legislation and institutional requirements. The participants provided their written informed consent to participate in this study.

## Author contributions

KL, DL, HP, MRF, and KT contributed to the conceptualization and design of the study. KL, DL, and HP collected data. JMR, DP, SK, and KL created the coding scheme and performed the coding. KL wrote the first draft of the manuscript. MFR, DP, and SK wrote sections of the manuscript. MS contributed further edits of the paper. MF revised the manuscript after reviewer comments. All authors contributed to revision, read, and approved the submitted version.

## References

[1] Nowland R Necka EA Cacioppo JT. Loneliness and social internet use: pathways to reconnection in a digital world? Persp Psychol Sci. (2018) 13:70–87. 10.1177/1745691617713052 28937910

[2] Rodríguez MD Gonzalez VM Favela J Santana PC. Home-based communication system for older adults and their remote family. Comput Human Behav. (2009) 25:609–18. 10.1016/j.chb.2008.08.017

[3] Cotten SR Ford G Ford S Hale TM. Internet use and depression among older adults. Comput Human Behav. (2012) 28:496–9. 10.1016/j.chb.2011.10.021

[4] Monahan C Macdonald J Lytle A Apriceno M Levy SR. COVID-19 and ageism: how positive and negative responses impact older adults and society. Am Psychol. (2020) 75:7. 10.1037/amp0000699 32672988

[5] Tyrrell CJ Williams KN. The paradox of social distancing: Implications for older adults in the context of COVID-19. Psychol Trauma. (2020) 12:S214–6. 10.1037/tra0000845 32525379

[6] Beller J Wagner A. Loneliness, social isolation, their synergistic interaction, and mortality. Health Psychol. (2018) 37:808–13. 10.1037/hea0000605 30138019

[7] Wenger GC Davies R Shahtahmasebi S Scott A. Social isolation and loneliness in old age: review and model refinement. Ageing Soc. (1996) 16:333–58. 10.1017/S0144686X00003457

[8] Chopik WJ. The benefits of social technology use among older adults are mediated by reduced loneliness. Cyberpsychol Behav Soc Netw. (2016) 19:551–6. 10.1089/cyber.2016.0151 27541746 PMC5312603

[9] Bradley N Poppen W. Assistive technology, computers and Internet may decrease sense of isolation for homebound elderly and disabled persons. Technol Disabil. (2003) 15:19–25. 10.3233/TAD-2003-15104

[10] Wilson G Gates JR Vijaykumar S Morgan DJ. Understanding older adults' use of social technology and the factors influencing use. Ageing Soc. (2023) 43:222–45. 10.1017/S0144686X21000490

[11] Wang HH Wu SZ Liu YY. Association between social support and health outcomes: a meta-analysis. Kaohsiung J Med Sci. (2003) 19:345–50. 10.1016/S1607-551X(09)70436-X 12926520 PMC11918198

[12] Tomaka J Thompson S Palacios R. The relation of social isolation, loneliness, and social support to disease outcomes among the elderly. J Aging Health. (2006) 18:359–84. 10.1177/0898264305280993 16648391

[13] Heinrich LM Gullone E. The clinical significance of loneliness: a literature review. Clin Psychol Rev. (2006) 26:695–718. 10.1016/j.cpr.2006.04.002 16952717

[14] Hawkley LC Cacioppo JT. Loneliness matters: a theoretical and empirical review of consequences and mechanisms. Ann Behav Med. (2010) 40:218–27. 10.1007/s12160-010-9210-8 20652462 PMC3874845

[15] Shankar A McMunn A Banks J Steptoe A. Loneliness, social isolation, and behavioral and biological health indicators in older adults. Health Psychol. (2011) 30:377–85. 10.1037/a0022826 21534675

[16] Piper A Breckenridge-Jackson I She's leaving home: a large sample investigation of the empty nest syndrome (2017). SOEPpaper No. 910. Available online at: https://ssrn.com/abstract=3169899

[17] Gerst-Emerson K Jayawardhana J. Loneliness as a public health issue: the impact of loneliness on health care utilization among older adults. Am J Public Health. (2015) 105:1013–9. 10.2105/AJPH.2014.302427 25790413 PMC4386514

[18] Armitage R Nellums LB. COVID-19 and the consequences of isolating the elderly. Lancet Pub Health. (2020) 5:5. 10.1016/S2468-2667(20)30061-X 32199471 PMC7104160

[19] González-Rodríguez A Labad J. Salud mental en tiempos de la COVID: reflexiones tras el estado de alarma. Med Clin. (2020) 155:392–4. 10.1016/j.medcli.2020.07.009 32958264 PMC7381887

[20] Giallonardo V Sampogna G Vecchio V Del Luciano M Albert U Carmassi C. The impact of quarantine and physical distancing following COVID-19 on mental health: study protocol of a multicentric Italian population trial. Front Psychiatry. (2020) 11:533. 10.3389/fpsyt.2020.00533 32581895 PMC7290062

[21] Kar SK Arafat SMY Sharma P Dixit A Marthoenis M Kabir R. COVID-19 pandemic and addiction: current problems and future concerns. Asian J Psychiatr. (2020) 51:102064. 10.1016/j.ajp.2020.102064 32305033 PMC7151310

[22] Sikali K. The dangers of social distancing: how COVID-19 can reshape our social experience. J Commun Psychol. (2020) 48:430. 10.1002/jcop.22430 32880991 PMC7461541

[23] Lewnard JA Lo NC. Scientific and ethical basis for social-distancing interventions against COVID-19. Lancet Infect Dis. (2020) 20:6. 10.1016/S1473-3099(20)30190-0 32213329 PMC7118670

[24] Nanda A Vura NVRK Gravenstein S. COVID-19 in older adults. Aging Clin Exp Res. (2020) 32:7. 10.1007/s40520-020-01581-5 32390064 PMC7211267

[25] Wu Z McGoogan JM. Characteristics of and important lessons from the coronavirus disease 2019 (COVID-19) outbreak in China. JAMA. (2020) 323:13. 10.1001/jama.2020.2648 32091533

[26] Saladino V Algeri D Auriemma V. The psychological and social impact of COVID-19: new perspectives of well-being. Front Psychol. (2020) 11:1–6. 10.3389/fpsyg.2020.577684 33132986 PMC7561673

[27] Gross EF Juvonen J Gable SL. Internet use and well-being in adolescence. J Soc Issues. (2002) 58:75–90. 10.1111/1540-4560.00249

[28] Valkenburg PM Peter J. The effects of instant messaging on the quality of adolescents' existing friendships: a longitudinal study. J Commun. (2009) 59:79–97. 10.1111/j.1460-2466.2008.01405.x

[29] Lee MA Ferraro KF Kim G. Digital technology use and depressive symptoms among older adults in Korea: beneficial for those who have fewer social interactions? Aging Ment Health. (2020) 2:1–9. 10.1080/13607863.2020.1839863 33131296

[30] Elliot AJ Mooney CJ Douthit KZ Lynch MF. Predictors of older adults' technology use and its relationship to depressive symptoms and well-being. J Gerontol B Psychol Sci Soc Sci. (2013) 69:667–77. 10.1093/geronb/gbt109 24304556 PMC4158393

[31] Sum S Mathews RM Hughes I Campbell A. Internet use and loneliness in older adults. Cyberpsychol Behav. (2008) 11:208–11. 10.1089/cpb.2007.0010 18422415

[32] Bruck L. Connecting: residents meet computers. Nursing Homes Long Term Care Manage. (2002) 3:22–8. 10.1525/ctx.2004.3.4.22

[33] Clark DJ. Older adults living through and with their computers. CIN. (2002) 20:117. 10.1097/00024665-200205000-00012 12021610

[34] Kato S Kato Y Usuki K. Associations between dependency on line text messaging and occurrence of negative emotions in line group chats. Adv Psychol Mental Health Behav Stu. (2019) 24:188–209. 10.4018/978-1-5225-9412-3.ch008

[35] Kraut R Patterson M Lundmark V Kiesler S Mukophadhyay T Scherlis W. Internet paradox: a social technology that reduces social involvement and psychological well-being? Am Psychol. (1998) 53:1017–31. 10.1037/0003-066X.53.9.1017 9841579

[36] NIE NH. Sociability, interpersonal relations, and the internet. Am Behav Sci. (2001) 45:420–35. 10.1177/00027640121957277

[37] Nie NH Hillygus DS Erbring L. Internet use, interpersonal relations and sociability: findings from a detailed time diary study. Int Everyday Life. (2002) 7:215–43. 10.1002/9780470774298.ch7

[38] Holman EA Grisham EL. When time falls apart: The public health implications of distorted time perception in the age of COVID-19. Psychol Trauma. (2020) 12:756. 10.1037/tra0000756 32525373

[39] Chen YRR Schulz PJ. The effect of information communication technology interventions on reducing social isolation in the elderly: a systematic review. J Med Int Res. (2016) 18:e18. 10.2196/jmir.4596 PMC4751336 26822073

[40] Age UK. Digital Inclusion Evidence Review. London: Age UK. (2018).

[41] Büchi M Just N Latzer M. Modeling the second-level digital divide: a five-country study of social differences in Internet use. New Media Soc. (2016) 18:2703–22. 10.1177/1461444815604154

[42] Government Digital Service. Policy Paper: Government Digital Inclusion Strategy. London: Government Digital Service (2014).

[43] Blank G Groselj D. Dimensions of internet use: amount, variety, and types. Inf Commun Soc. (2014) 17:417–35. 10.1080/1369118X.2014.889189

[44] van Deursen AJAM Helsper EJ. The third-level digital divide: who benefits most from being online? Commun Inf Technol. (2015) 14:29–52. 10.1108/S2050-206020150000010002

[45] Scheerder A van Deursen A van Dijk J. Determinants of Internet skills, uses and outcomes. A systematic review of the second- and third-level digital divide. Telematic Inf. (2017) 34:1607–24. 10.1016/j.tele.2017.07.007

[46] Flandorfer P. Population ageing and socially assistive robots for elderly persons: the importance of sociodemographic factors for user acceptance. Int J Popul Res. (2012) 2012:1–13. 10.1155/2012/829835

[47] Søraa RA Tøndel G Kharas MW Serrano JA. What do older adults want from social robots? A qualitative research approach to human-robot interaction (HRI). Studies Int J Soc Robot. (2023) 15:411–24. 10.1007/s12369-022-00914-w

[48] Lee HR Šabanović S Chang WL Nagata S Piatt J Bennett C. Steps toward participatory design of social robots. In: Proceedings of the 2017 ACM/IEEE International Conference on Human-Robot Interaction. New York, NY, USA: ACM (2017), 244–53.

[49] Smith A. Older Adults Technology Use, Pew Research Center: Internet, Science & Tech. United States of America (2014). CID: 20.500.12592/95zsg4. Retrieved from: https://policycommons.net/artifacts/620165/older-adults-and-technology-use/1601368/ (accessed September 22, 2023).

[50] Fearn M Harper R Major G Bhar S Bryant C Dow B. Befriending older adults in nursing homes: volunteer perceptions of switching to remote befriending in the COVID-19 era. Clin Gerontol. (2021) 44:1–9. 10.1080/07317115.2020.1868646 33403935

[51] Fraune MR Langlois D Au RHY Preusse H Rheman M Ling K. With Age Comes Technology Wisdom: Across Cultures, the Effect of Affinity for Technology on Group Cohesion Mental Health During Social Isolation Depends on Age (2022). Available online at: https://ssrn.com/abstract=4049530

[52] Connelly K ur Rehman Laghari K Mokhtari M Falk TH. Approaches to understanding the impact of technologies for aging in place: a mini-review. Gerontology. (2014) 60:282–8. 10.1159/000355644 24457288

[53] Peek STM Luijkx KG Rijnaard MD Nieboer ME van der Voort CS Aarts S. Older adults' reasons for using technology while aging in place. Gerontology. (2015) 62:226–37. 10.1159/000430949 26044243

[54] Morse JM. The significance of saturation. Qual Health Res. (1995) 5:147–9. 10.1177/104973239500500201

[55] Sandelowski M. Sample size in qualitative research. Res Nurs Health. (1995) 18:179–83. 10.1002/nur.4770180211 7899572

[56] Hennink M Kaiser BN. Sample sizes for saturation in qualitative research: a systematic review of empirical tests. Soc Sci Med. (2022) 292:114523. 10.1016/j.socscimed.2021.114523 34785096

[57] Šabanović S Chang WL Bennett CC Piatt JA Hakken D. A robot of my own: participatory design of socially assistive robots for independently living older adults diagnosed with depression. In: Human Aspects of IT for the Aged Population. Design for Aging: First International Conference, ITAP 2015, Held as Part of HCI International 2015, Los Angeles, CA, USA. (2015), 104–14.

[58] Azenkot S Feng C Cakmak M. Enabling building service robots to guide blind people a participatory design approach. In: 2016 11th ACM/IEEE International Conference on Human-Robot Interaction (HRI). Piscataway, NJ: IEEE (2016), p. 3–10.

[59] King N Horrocks C Brooks J. Interviews in Qualitative Research. 2nd Edn. London: Sage Publications Ltd. (2019).

[60] Saldana J. The Coding Manual for Qualitative Researchers. London: Sage Publications Ltd (2021).

[61] Ihm J Hsieh YP. The implications of information and communication technology use for the social well-being of older adults. Inf Commun Soc. (2015) 18:1123–38. 10.1080/1369118X.2015.1019912

[62] Schouten AP Portegies TC Withuis I Willemsen LM Mazerant-Dubois K. Robomorphism: Examining the effects of telepresence robots on between-student cooperation. Comput Hum Behav. (2022) 126:106980. 10.1016/j.chb.2021.106980

[63] Bos N Olson J Gergle D Olson G Wright Z. Effects of four computer-mediated communications channels on trust development. In: Proceedings of the SIGCHI Conference on Human Factors in Computing Systems. New York, NY, USA: ACM (2002), p. 135–40.

[64] Okdie BM Guadagno RE Bernieri FJ Geers AL Mclarney-Vesotski AR. Getting to know you: Face-to-face versus online interactions. Comput Hum Behav. (2011) 27:153–9. 10.1016/j.chb.2010.07.017

[65] Fraune MR Wilson E Loya J Tsui KM. Two is better than one: telepresence team members feel closer to the group when there are multiple telepresence members, and they engage in task rather than social activities. Trans Hum Robot Interact. (under review).

[66] Trope Y Liberman N. Construal-level theory of psychological distance. Psychol Rev. (2010) 117:440–63. 10.1037/a0018963 20438233 PMC3152826

[67] Abdi J Al-Hindawi A Ng T Vizcaychipi MP. Scoping review on the use of socially assistive robot technology in elderly care. BMJ Open. (2018) 8:e018815. 10.1136/bmjopen-2017-018815 29440212 PMC5829664

[68] Shibata T Wada K. Robot therapy: a new approach for mental healthcare of the elderly – a mini-review. Gerontology. (2011) 57:378–86. 10.1159/000319015 20639620

[69] Block AE Christen S Gassert R Hilliges O Kuchenbecker KJ. The Six Hug Commandments: Design and Evaluation of a Human-Sized Hugging Robot with Visual and Haptic Perception. In: 2021 16th ACM/IEEE International Conference on Human-Robot Interaction (HRI), Piscataway, NJ: IEEE (2021), p. 380–8.

[70] DiSalvo C Gemperle F Forlizzi J Montgomery E. The Hug: an exploration of robotic form for intimate communication. In: The *12th IEEE International Workshop on Robot and Human Interactive Communication, 2003 Proceedings ROMAN*. Piscataway, NJ: IEEE (2003), p. 403–8.

[71] Au RHY Ling K Fraune MR Tsui KM. Robot Touch to Send Sympathy: Divergent Perspectives of Senders and Recipients. In: 2022 17th ACM/IEEE International Conference on Human-Robot Interaction (HRI). Piscataway, NJ: IEEE (2022), p. 372–82.

[72] Kurniawan S Zaphiris P. Research-derived web design guidelines for older people. In: Proceedings of the 7th International ACM SIGACCESS Conference on Computers and Accessibility. New York, NY, USA: ACM (2005), p. 129–35.

[73] Tsui KM. The development of telepresence robots for people with disabilities (Doctoral dissertation). University of Massachusetts Lowell (2014).

[74] Greenhalgh T Wherton J Papoutsi C Lynch J Hughes G A'Court C. beyond adoption: a new framework for theorizing and evaluating non-adoption, abandonment, and challenges to the scale-up, spread, and sustainability of health and care technologies. J Med Internet Res. (2017) 19:e367. 10.2196/jmir.8775 29092808 PMC5688245

[75] Boudouraki A Fischer JE Reeves S Rintel S. “I can't get round” recruiting assistance in mobile robotic telepresence. In: Proceedings of the ACM on Human-computer Interaction, 4(CSCW3) (2021). p. 1–21.36644216

[76] Ling K Langlois D Preusse H Rheman M Parson D Kuballa S. If you weren't connected to the Internet, you were not alive: experience of using social technology during COVID-19 in adults 50+. Front Pub Health. (2023) 10.3389/fpubh.2023.1177683 PMC10590895 37876716

[77] Slate JR Manuel M Jr KHB. The “Digital Divide”: Hispanic college students' views of educational uses of the Internet. Assess Eval High Educ. (2002) 27:75–93. 10.1080/02602930120105081

[78] Reddick CG Enriquez R Harris RJ Sharma B. Determinants of broadband access and affordability: an analysis of a community survey on the digital divide. Cities. (2020) 106:102904. 10.1016/j.cities.2020.102904 32921864 PMC7480260

[79] Katz VS Levine MH. Connecting to learn: promoting digital equity among america's hispanic families. In: Joan Ganz Cooney Center at Sesame Workshop. New York, NY (2015).

[80] Matthews K Nazroo J Marshall A. Digital inclusion in later life: cohort changes in internet use over a ten-year period in England. Ageing Soc. (2019) 39:1914–32. 10.1017/S0144686X18000326

[81] Gracia E Herrero J. Internet use and self-rated health among older people: a national survey. J Med Internet Res. (2009) 11:e49. 10.2196/jmir.1311 19955041 PMC2802559

[82] Neves BB Amaro F Fonseca JRS. Coming of (Old) age in the digital age: ICT usage and non-usage among older adults. Sociol Res. (2013) 18:22–35. 10.5153/sro.2998

[83] Yu RP Ellison NB McCammon RJ Langa KM. Mapping the two levels of digital divide: Internet access and social network site adoption among older adults in the USA. Inf Commun Soc. (2016) 19:1445–64. 10.1080/1369118X.2015.1109695

[84] Bergström A. Digital equality and the uptake of digital applications among seniors of different age. Nordicom Rev. (2017) 38:79–91. 10.1515/nor-2017-0398

[85] Cresci MK Jarosz PA. Bridging the digital divide for urban seniors: community partnership. Geriatr Nurs. (2010) 31:455–63. 10.1016/j.gerinurse.2010.10.006 21188756

[86] Beal FM. Double jeopardy. Meridians. (2008) 8:166–76. 10.2979/MER.2008.8.2.166

[87] Hill Collins P. Black Feminist Thought. London: Routledge. (2002).

[88] Suntai Z Beltran SJ. The intersectional impact of race/ethnicity and sex on access to technology among older adults. Gerontologist. (2022) 63:1162–71. 10.1093/geront/gnac178 36477498

[89] Liu BCP. The impact of intersectionality of multiple identities on the digital health divide, quality of life and loneliness amongst older adults in the UK. The Br J Soc Work. (2021) 51:3077–97. 10.1093/bjsw/bcaa149

[90] Fang ML Wong KLY Remund L Sixsmith J Sixsmith A. Technology access is a human right! Illuminating intersectional, digital determinants of health to enable agency in a digitized era. In: TMS Proceedings 2021. Veinna: American Psychological Association (2021).

[91] Bardzell S. Feminist HCI. In: Proceedings of the SIGCHI Conference on Human Factors in Computing Systems. New York, NY, USA: ACM (2010), p. 1301–10.

[92] Miranda L Castellano G Winkle K. Examining the State of Robot Identity. In: Companion of the 2023 ACM/IEEE International Conference on Human-Robot Interaction. New York, NY, USA: ACM (2023), p. 658–62.

[93] Khosravi P Rezvani A Wiewiora A. The impact of technology on older adults' social isolation. Comput Human Behav. (2016) 63:594–603. 10.1016/j.chb.2016.05.092

