# Supplementary material for: “If you weren't connected to the Internet, you were not alive”: experience of using social technology during COVID-19 in adults 50+
Source: Front Public Health. 2023 Oct 9;11:1177683. doi: 10.3389/fpubh.2023.1177683 (PMC10590895; doi:10.3389/fpubh.2023.1177683)
Supplement: Supplementary file 1 [file Table_1.DOCX]

**Appendix A**

Interview Outline

**Warm up (5-10 mins)**

Hi, I’m *X*, how are you? Did you have any problems getting on this call?

Here’s the link to the consent form for this research study; I’ve put it in the Zoom chat box.

We would like to record this interview so that we can refer back to it, do I have consent to record this interview?

In this interview, we are asking you some questions about your technology use and opinions, specifically in the context of Covid-19. The questions will cover your social experiences with Covid-19, your current technology use, and how technology could help you socialize. Are you ready to begin?

What activities did you do while social distancing? What did you find enjoyable then?

Were you doing these activities by yourself or with others in some way?

Also, what is your social life like? How has it changed since Covid-19?

**Technology (10 min)**

In the survey/Just now, you said that you used _____ to socialize, what do you think about that platform/item/etc?

What is going wrong/is difficult? What is not allowed for? How do we solve problems?

(If screen time is not mentioned) One issue that has come up multiple times is the excessive screen use required for much of our existing technology, is this an issue for you?

How has tech use changed? Is this change permanent?

What technology are you interested in/do you want for socialization? How should/would it benefit your social life?

Who have you grown apart from during the pandemic? Why?

How is your level/amount of contact different with people lacking certain tech/with a poorer understanding of tech than others?

Did you purchase new technology/start learning to use new tech during this time? Why hadn’t you owned this tech before?

What would convince you to buy a robot/new tech?

**Robots (20 min)**

We want to think about future technologies beyond just screens. One possibility is the use of robots. We would like to try and create robotic technology to facilitate communication/social interaction.

*(Facilitator shares a selection of video clips that give examples of robots behaving socially via screen share)*

We are going to watch a selection of video clips of different robots, I’ll be giving short descriptions of these clips.

Paro is a stuffed seal robot that moves and makes soothing sounds. It is used in therapy, usually with people in nursing homes.

Jibo is a personal assistant robot that can do tasks like share recipes, hold simple conversations, and dance.

Pepper is a more advanced robot that can interact both with conversation and its touch screen tablet. It can also recognize face and emotions.

This is Misty II, a recently introduced robot platform. Misty can navigate around rooms, recognize faces, respond to touch and sound, and be connected to other types of technology (like the Amazon Alexa, for example).

This is a NAO, a robot that can be programed to do a variety of tasks, including singing songs, dancing, and playing games.

This is one of Boston Dynamics’ dog robots (Spot), which can maneuver across multiple types of terrain and perform a variety of tasks.

This is Dragonbot, a robot that reads stories to children.

Now that you have seen these videos, I am going to ask you a series of questions, and I want you to think about how robots might assist.

If social isolation continued for five years, what would you want your robotic technology to do in order to facilitate social contact with other people?

What social interactions are most rewarding? For example, a forum you are a member of, versus a group of friends you meet for lunch. *[if there is uncertainty]:* Could you remind me if the group you are talking about is online only or not?

For groups like that that made you feel more connected, how could a robot help?

Can you think of any benefits of robots vs screens for your social life? What challenges do you see for robots vs screens?

Can you think of any benefits of robots to your social life? What barriers do you see in terms of robots joining your social life?

**Personas/Categories (10 mins)**

Are you quicker to try new technology than your friends?

How involved do you prefer your technology in day-to-day life? (Like, what level of notifications/beeps are you comfortable with per day)

What kind of social involvement do you have/want in your friends’/family’s/etc lives

What (social?) tasks would you have robots do?

How would you want to interact with it (superior/inferior/toy/pet/peer)?

If I were to wave a magic wand and give you a robot, what would you want it to be able to do? (tech possible in the next few years)

**Goodbye**

We’re coming to a close; do you have any final thoughts or anything else you wanted to tell us?

Thank you so much for participating in this interview! We will be emailing you shortly with the code for your $30 Amazon gift card.

We’re conducting a third study, which will be a group interview. It will focus on designing social robots. The group interview would take about 90 minutes, and you would be compensated with a $70 Amazon gift card. Are you interested in participating in the group interview study?

Great! We would like to mail you a packet of materials that you will use during the group interview. We will include a prepaid return shipping label for you to send the materials back to us. Are you comfortable with sharing a mailing address with us?

We will email you to schedule for the group interview within the next few weeks. Do you have any questions about the group interview study?

Sounds good, thank you again for your participation. If you have any further questions or concerns, please contact us at covidsocialgroups@gmail.com. I hope you have a nice day, good bye!
