# Supplementary material for: “If you weren't connected to the Internet, you were not alive”: experience of using social technology during COVID-19 in adults 50+
Source: Front Public Health. 2023 Oct 9;11:1177683. doi: 10.3389/fpubh.2023.1177683 (PMC10590895; doi:10.3389/fpubh.2023.1177683)
Supplement: Supplementary file 2 [file Table_2.DOCX]

**Appendix B**

Coding Framework

| Code | Operational Definition | Example |
| --- | --- | --- |
| Introversion | Participants describes themself as "introverted" or "an introvert" | "I was kind of an introvert" |
| High social involvement | Participant expresses a desire for high social involvement; spending a lot of time with friends or family | "I'm very social. So yeah, maybe not the same person all the time, but I'm talking with people in my life every day." |
| Low social involvement | Participant expresses a preference for low or occasional social interactions with friends or family | "We check in with each other… once every couple months" |
| Conversations | Participant expresses a preference for social interactions in which they can have a meaningful conversation | "it has to be something that allows us to talk" |
| Face-to-face | Participant expresses a preference for face-to-face social interactions, as opposed to online or phone interactions | "I prefer face-to-face, one-on-one" |
| Variety | Participants expresses a desire to have multiple modes of social interaction in their life; e.g. both face-to-face and online | "all valuable, but in different ways... I can't really evaluate them and compare them to each other because they're very different activities" |
| Impact on parent relationship | Participant mentions that the COVID-19 pandemic had an impact on their relationship with a parent | "my mother, who lives in Wisconsin, I haven't been able to see her" |
| Impact on friends relationship | Participant mentions that the COVID-19 pandemic had an impact on their relationship with a friend or friends | "there were some not so close friends that I stopped seeing" |
| Impact on coworker relationship | Participant mentions that the COVID-19 pandemic had an impact on their relationship with coworkers | "[I've grown apart from] my business associates" |
| Impact on family relationship | Participant mentions that the COVID-19 pandemic had an impact on their relationship with a non-parent family member | "We have two older kids... Marie doesn't like technology, and so if you don't see Marie in person, you don't see Marie" |
| Decrease in social interaction | Participant describes an overall decrease in level of social interactions due to the COVID-19 pandemic | "it's been cut down to nearly nothing, as far as social" |
| No change in social interaction | Participant describes that there was no changes in their overall level of social interaction to the COVID-19 pandemic | "even with the the social distancing... it's not made a whole lot of differences in [social] stuff that we do" |
| Risk of infection | Participant describes risk of infection as a reason for altering their social behavior | "there's a lot of friends that I would like to see, but I don't want to endanger them" |
| Lack of casual interactions | Participant discusses a decrease in the amount of casual or incidental social interactions, such as with coworkers or neighbors | "without realizing it, growing apart from my local friends because I'm just not seeing them and interacting with them" |
| High technology involvement | Participant expresses a preference for a high level of social technology involvement in their everyday life, such as using it throughout the day or having a high level of notifications | "there is no day or no time that I am [away from it]" |
| Low technology involvement | Participant expresses a preference for a low level of social technology involvement in their everyday life, such as preferring to only use social technology occasionally or having few to no notifications | "I usually turn them all off. I turn off every notification I can" |
| Conflicted regarding technology involvement | Participant expresses that their preferences for level of social technology involvement varies, depends on the technology or the person, or expresses conflicted feelings regarding social technology involvement | "I want to be notified, but I don't want to be notified" |
| Quick to adopt new social technology | Participant self-describes as an "early adopter" or person who is quick to try new social technology | "quicker [to adopt new social technology]... if I had if I had all the money in the world, I would buy gadgets and gizmos" |
| Slow to adopt new social technology | Participant self-describes as slow to try new social technology | "no, I was way behind the curve on smartphones" |
| Variable adaption rate of new technology | Participant states that how quick they are to try new technology can vary, or depends on other factors | "it would depend on what it is" |
| Desire for new social technology | Participant expresses a desire to acquire new social technology | "I've been looking at the Iphone watch thing" |
| No desire for new social technology | Participant states that they do not wish to purchase or acquire any new social technology | "nothing [new] really jumps out at me... the technology seems to be pretty solid" |
| Desire for existing social technology to be improved | Participant expresses a desire that they would rather their existing social technology be improved, as opposed to acquiring a new social technology | "It should be easier to bring up" |
| Switch from in-person to online interaction | Participant describes a relationship or social group which previously existed in-person transitioning to online or remote due to the COVID-19 pandemic | "I used to get together with some friends… the board game group is now just online" |
| Increase in social technology use | Participant describes an overall increase in their level of social technology use due to the COVID-19 pandemic | "I definitely use tech more" |
| Adaption of video call technology | Participant discusses trying video call technology for the first time during the COVID-19 pandemic, or significantly increasing their use of video call technology during this time | "Zoom meeting stuff is new to me" |
| Permanent change in social technology use | Participant expresses a belief that their changes in social technology use during the COVID-19 pandemic will become permanent after pandemic regulations decrease | "I think there are permanent changes to this" |
| Gratitude for social technology | Participant expresses gratitude for social technology allowing them to have social interactions | "It has a purpose... touching base with some old friends, keeping up with faraway friends, even keeping up with friends that are close" |
| Benefits of video call technology | Participant discusses a way in which they believe that video call technology is superior to other forms of social technology, e.g. because you can see body language and facial expressions | "I like [that] ... I can see you... I can sort of read your expressions" |
| Difficulty learning to use social technology | Participant expresses that they themselves have difficulty learning how to use new social technology | "I sometimes get confused [with] ... who's calling who and how to access it... where to click the correct place" |
| Difficulty of parents or friends to learning how to use social technology | Participant expresses that a friend or family member has difficulty learning how to use new social technology | "if you're not there to show her how you did something… she doesn't retain how to how to use it" |
| Internet access issues | Participant describes difficulty gaining access to the internet, such as a lack of broadband in their area | "a lot of my clients do not have access to internet" |
| Glitches in social technology | Participant describes challenges with social technology glitching or not working as it should | "sometimes these things will launch with no problems, and sometimes, you know, I can't make them work to save my life" |
| Difficulty with amount of screen time | Participant states that the amount of time spent looking at a screen is an issue for them | "my eyesight kind of went down a little bit, from looking at the screen" |
| No difficulty with amount of screen time | Participant states that the amount of time spent looking at a screen is not an issue for them | "all night long and stare at computer screens and it's really not a problem" |
